# Unveiling Immune Response Mechanisms in Mpox Infection Through Machine Learning Analysis of Time Series Gene Expression Data

**DOI:** 10.3390/life15071039

**Published:** 2025-06-30

**Authors:** Qinglan Ma, Xianchao Zhou, Lei Chen, Kaiyan Feng, Yusheng Bao, Wei Guo, Tao Huang, Yu-Dong Cai

**Affiliations:** 1 School of Life Sciences, Shanghai University, Shanghai 200444, China; mql1117@shu.edu.cn (Q.M.); bys@shu.edu.cn (Y.B.); 2 Center for Single-Cell Omics, School of Public Health, Shanghai Jiao Tong University School of Medicine, Shanghai 200025, China; zhouxianchao@sjtu.edu.cn; 3 College of Information Engineering, Shanghai Maritime University, Shanghai 201306, China; lchen@shmtu.edu.cn; 4 Department of Computer Science, Guangdong AIB Polytechnic College, Guangzhou 510507, China; kyfeng@gdaib.edu.cn; 5 Shenzhen Institute of Advanced Technology, Chinese Academy of Sciences, Shenzhen 518055, China; gw_1992@sjtu.edu.cn; 6 Bio-Med Big Data Center, CAS Key Laboratory of Computational Biology, Shanghai Institute of Nutrition and Health, University of Chinese Academy of Sciences, Chinese Academy of Sciences, Shanghai 200031, China; 7 Department of Artificial Intelligence and Digital Health, CAS Engineering Laboratory for Nutrition, Shanghai Institute of Nutrition and Health, University of Chinese Academy of Sciences, Chinese Academy of Sciences, Shanghai 200031, China

**Keywords:** monkeypox virus, immune, machine learning, classification rule

## Abstract

Monkeypox virus (Mpox) has recently drawn global attention due to outbreaks beyond its traditional endemic regions. Understanding the immune response to Mpox infection is essential for improving disease management and guiding vaccine development. In this study, we used several machine learning algorithms to analyze time series gene expression data from macaques infected with Mpox, aiming to uncover key immune-related genes involved in different stages of infection. The dataset covered early infection, late infection, and rechallenge phases. We applied nine feature ranking methods to analyze the feature importance, obtaining nine feature lists. Then, the incremental feature selection method was applied to each list to extract key genes and build efficient prediction models and classification rules for each list. This procedure employed twelve classification algorithms and the Synthetic Minority Oversampling Technique. Key genes—such as CD19, MS4A1, and TLR10—were repeatedly identified from multiple feature lists, and are known to play vital roles in B-cell activation, antibody production, and innate immunity. Furthermore, we identified several novel key genes (HS3ST1, SPAG16, and MTARC2) that have not been reported previously. These findings offer valuable insights into the host immune response and highlight potential molecular targets for monitoring and intervention in Mpox infections.

## 1. Introduction

Monkeypox virus (Mpox) is a double-stranded DNA virus belonging to the genus Orthopoxvirus and has been highlighted recently as a significant global health threat [1]. Mpox was traditionally confined to West and Central Africa but has been seen in several non-endemic regions since 2017, with a rapid uptick in cases in 2022 [2,3]. This provides a reason to consider that such a rapid geographic extension and increase in human infections underscore the potential of zoonotic transmission by Mpox and resultant surveillance for control. During initial postinfection, following entry via either the respiratory or the skin portal, Mpox virus localizes to initial sites of infection, including mucosa and epithelium of the oral and respiratory tracts. Later on, it involves lymph nodes due to viremia, and the involved lymph organs are the tonsils, spleen, and liver. Also, through secondary viremia, when it disseminates to the lungs, kidney, intestines, and skin, clinical symptomatology is brought out [4,5,6,7].

Both innate and adaptive immunity are involved in the host immune response. Interleukins and chemokines (such as CCL2 and CCL5) are highly upregulated during innate immunity, while tumor necrosis factor-α and interferon-γ levels are suppressed [8,9]. Mpox exhibits its immune evasion from the host by inhibiting the TLR signaling pathways and interferon signaling. Infection strongly upregulates the TLR pathways, NF-κB, and leukocyte migration-related genes [10]. Pathological investigations have revealed multi-organ inflammation related to Mpox; most inflammation manifests in the form of infiltration of inflammatory cells, along with a collection of viral antigens [7,11,12]. The infection induces adaptive immunity, comprising IgM and IgG antibody responses, along with proliferation of both CD4+ and CD8+ T cells [13,14,15,16]. The virus modulates the host immune response by suppression of T-cell activation, allowing persistence. The host mounts specific antibodies and memory B and T cells in an immunological memory response. Individuals vaccinated against smallpox have a superior IgG response postinfection [17,18].

Given that our analysis is based on Mpox-infected macaque models, it is important to reflect on the translational relevance of these findings to human infection. Rhesus macaques are widely used in orthopoxvirus research due to their close evolutionary and immunological similarity to humans [19]. Comparative studies have demonstrated consistent activation of innate and adaptive immune pathways—including cytokine secretion profiles, B-cell activation, and T-cell memory formation—in both species upon infection [16,20,21]. Although interspecies differences in gene regulation and response timing exist, macaque models provide a robust proxy for dissecting host–pathogen interactions and identifying conserved immunological markers, particularly in the absence of extensive human time series transcriptomic data.

To further explore these immunological mechanisms, especially across distinct infection stages and in high-dimensional datasets, machine learning (ML) has emerged as a powerful tool in biomedical research. It is capable of efficiently processing complex, large-scale biological data and uncovering latent patterns that traditional statistical methods may overlook [22]. In the infectious diseases context, different ML techniques are used to forecast disease outcomes, identify biomarkers, and help improve diagnostic accuracy [23]. Through analyzing large-scale biological data, ML can offer valuable insights into the interactions between host and pathogen and into the immune response, and may highlight putative targets for therapeutic intervention [24]. For example, a deep convolutional neural network model was developed to recognize skin lesions caused by Mpox and thus assist in its early detection to stem the outbreak of the infection [25]. Stern et al. used ML methods during serological multiplex assays to differentiate antibody responses against Mpox and related smallpox vaccines with accuracy [26]. In the study of the Mpox infection, ML describes the features of different disease stages with precision and, finally, contributes to improved clinical management strategies [27]. However, previous studies adopted limited ML algorithms. As each algorithm has limitations, some essential information cannot be uncovered.

In this study, we employed several ML algorithms, including nine feature ranking methods, incremental feature selection (IFS) [28], the Synthetic Minority Oversampling Technique (SMOTE) [29], and twelve classification algorithms, to design an ML-based analysis framework. The above ML algorithms were properly integrated to extensively analyze a given dataset, mining essential information as much as possible. The ML-based analysis framework was applied to the gene expression characteristics of Mpox infection at three distinct stages, namely early challenge—which covers days 1–14—late challenge—which covers days 21–28—and finally rechallenge—which includes days 29–38—in rhesus macaques. The dataset is based on an Mpox inoculation experiment on 18 rhesus macaques with RNA expression profiles obtained from peripheral blood samples collected multiple times in the course of the study. The immune status at these three stages has been described in detail in the source article [30]. During the early challenge (days 1–14), viral replication is active, with rising plasma viral loads and significant clinical symptoms, including extensive skin lesions and systemic effects. During the late challenge (days 21–28), there is a decline in viral replication, an improvement in clinical symptoms, and the beginning of adjustment in the immune response, though the levels of antibodies continue to rise. During the rechallenge phase, days 29–38, protective immunity was observed, as all recovered animals showed high resistance to reinfection and maintained high levels of antibodies. The ML-based analysis framework first applied nine ranking methods to yield feature ranking lists. Then, they were fed into the IFS [28] method to extract essential features. This procedure employed twelve classification algorithms to evaluate the importance of feature subsets and SMOTE [29] to tackle the data imbalance problem. The intersection of essential features extracted from nine feature lists was analyzed, resulting in finding the most essential features, including some genes (CD19, MS4A1, and TLR10) related to B-cell activation, antibody production, and innate immunity, and novel ones (HS3ST1, SPAG16, and MTARC2). Furthermore, classification rules yielded by decision tree (DT) [31] were obtained, indicating the special expression patterns for different stages of Mpox infection. The goal of this study is to identify specific markers of Mpox infection progression and control based on an analysis of the immune response and dynamics between host and pathogen at each stage. The findings of this study are helpful for better therapeutic interventions and understanding of the host immune response against Mpox.

## 2. Materials and Methods

### 2.1. Data on Mpox Infection in Rhesus Macaques

This study obtained gene expression data of a group of macaques infected with monkeypox from the Aid et al. study [30]. The data was divided into three distinct classes: 1–14 days from the initial exposure (challenge-early), 21–28 days post-exposure (challenge-late), and 29–38 days during the rechallenge phase (rechallenge). Specifically, 112 samples were in the challenge-early group, 43 samples were in the challenge-late group, and 76 samples were in the rechallenge group. Each sample contained 35,611 gene features.

### 2.2. Feature Ranking Methods Used to Rank Features in Order of Importance

To uncover the specific gene features associated with Mpox infection, we utilized nine different feature ranking methods, including Categorical Boosting (CATBoost) [32], Least Absolute Shrinkage and Selection Operator (LASSO) [33], Extremely Randomized Trees (ExtraTrees) [34], Light Gradient Boosting Machine (LightGBM) [35], Monte Carlo Feature Selection (MCFS) [36], Random Forest (RF) [37], SelectKBest (SKB) [38], Ridge Regression (Ridge) [39], and eXtreme Gradient Boosting (XGBoost) [40]. All gene features can be widely evaluated using these feature ranking methods as they were designed using different principles. Their brief descriptions are provided in File S1.

### 2.3. Incremental Feature Selection

IFS is a method in ML for feature selection. This approach, in a systematic way, adds features into the model, assesses their impact on model performance, and derives the optimal feature subset that provides the best model performance [28]. IFS typically follows these steps: (1) Initialization: Start with an empty set of features. The features are ranked according to a predefined criterion such as feature importance. (2) Incremental addition: At each step, add one feature (the top feature from those not already selected) to the current set of features. (3) Model evaluation: Use the newly formed feature set to train the model, and use a predefined metric (for example, accuracy, F1 score [41,42,43,44,45,46], AUC [47,48,49,50]) to evaluate its performance. (4) Repeat steps 2 and 3 until all features are added or adding more features will not significantly improve the performance of the model. (5) Determine the optimal feature subset: Select the feature subset that has the best performance as the optimal feature subset. The model using the optimal feature subset is called the optimal model.

### 2.4. Synthetic Minority Oversampling Technique

SMOTE is a statistical method to solve the problem of class imbalance in ML datasets [29]. Introduced by Chawla et al. in 2002, it enhances the performance of prediction models by balancing the class distribution through creating artificial samples in the minority class. Unlike traditional oversampling with replacement, SMOTE generates synthetic and new samples. For a randomly selected sample from the minority class, SMOTE identifies its *k*-nearest neighbors in the same class, where *k* is a user-defined parameter. It creates a synthetic sample by interpolating between a randomly selected neighbor and the original sample. This synthetic sample is put into the minority class to enlarge its size. The process is repeated until the class distribution is balanced; the required oversampling level can be specified by the user, usually expressed as a percentage of the original size of the minority class.

### 2.5. Classification Algorithms

To execute the IFS method, it is essential to anchor it with algorithms from the realm of supervised classification. Accordingly, 12 classification algorithms were employed in this study. Among the above nine feature ranking methods, five of them (Ridge, ExtraTrees, LightGBM, RF, and XGBoost) were also adopted as classification algorithms. The other seven classification algorithms included Nearest Centroid Classifier (Ncentroid) [51], Stochastic Gradient Descent (SGD) [52], DT [31], Support Vector Machine (SVM) [53], Naïve Bayes Classifier (Bayes) [54], Adaptive Boosting (Adaboost) [55], and K-Nearest Neighbors (KNN) [56]. The brief descriptions of the above seven classification algorithms are also available in File S1.

### 2.6. Performance Evaluation

Weighted F1 is an extremely significant metric in ML, particularly when dealing with class imbalances [57]. It differs from macro F1, which is the average of F1 scores on all classes, in the sense that class sizes are taken into account and larger classes get heavier weights. It gives a precise evaluation of model performance under real-world settings and hence holds value for usage such as in medical diagnosis and detecting fraud, where class imbalanced data is the norm and has to be properly evaluated. The specific formula for this metric is as follows:

(1) $${Precision}_{i}=\frac{{TP}_{i}}{{TP}_{i}+{FP}_{i}},$$

(2) $${Precision}_{weighted}=\sum_{i=1}^{L}{Precision}_{i}\times w_{i},$$

(3) $${Recall}_{i}=\frac{{TP}_{i}}{{TP}_{i}+{FN}_{i}},$$

(4) $${Recall}_{weighted}=\sum_{i=1}^{L}{Recall}_{i}\times w_{i},$$

(5) $$Weighted\;F1=\frac{2\cdot {Precision}_{weighted}\cdot {Recall}_{weighted}}{{Precision}_{weighted}+{Recall}_{weighted}},$$

In the above formulas, i  denotes one individual class, $w_{i}\;$ is the proportion of samples in that class relative to the overall samples, and L indicates the total number of classes. Additionally, *TP* is an abbreviation for true positives, *FP* means false positives, and *FN* designates false negatives.

In addition, we further employed classic metrics, accuracy (ACC) and the Matthews correlation coefficient (MCC) [58,59], to display the performance of prediction models. ACC is one of the most widely used metrics, and is defined as the proportion of correctly predicted samples. MCC is more accurate than ACC when the dataset is imbalanced. Two matrices, *X* and *Y*, are constructed first, where *X* stores the true classes of all samples and *Y* collects the predicted classes of samples. Then, MCC can be computed as

(6) $$\mathrm{MCC}=\frac{cov(X,Y)}{\sqrt{cov(X,X)\cdot cov(Y,Y)}},$$

where cov(·,·) represents the correlation coefficient of two matrices.

### 2.7. Construction of the PPI Network

For the identified essential genes, we adopted the protein–protein interaction (PPI) network to analyze them. In the current study, protein interaction was retrieved and visualized based on the STRING v12.0 database. The STRING database gives an overview of functional protein relation and supports the examination of genetic networks. The PPI network retrieved was visualized based on Cytoscape (v3.10.1), a software platform with an open source that is capable of integrating, visualizing, and analyzing molecular interaction data. Each node’s degree was visualized in different colors in Cytoscape visualization [60].

### 2.8. Biological Function Enrichment

For the identified essential genes, enrichment analyses were carried out in R using the clusterProfiler package (v4.6.0) [61] with human gene annotation from org.Hs.eg.db (v3.18.0) [62]. The official gene symbols were used directly for gene ontology (GO) enrichment across biological process (BP), molecular function (MF), and cellular component (CC) ontologies, under Benjamini–Hochberg correction (adjusted *p* < 0.05). For Kyoto Encyclopedia of Genes and Genomes (KEGG) pathway analysis, the same symbols were first mapped to Entrez identifiers and then tested with the same correction scheme, excluding disease-related pathways. For both GO and KEGG analyses, enriched terms are first ranked by their Benjamini–Hochberg-adjusted *p*-values, the top fifteen terms are then selected, and the results are visualized as bubble plots in which each bubble’s size and color reflect the number and proportion of input genes involved and the strength of enrichment.

## 3. Results

**Figure 1:**
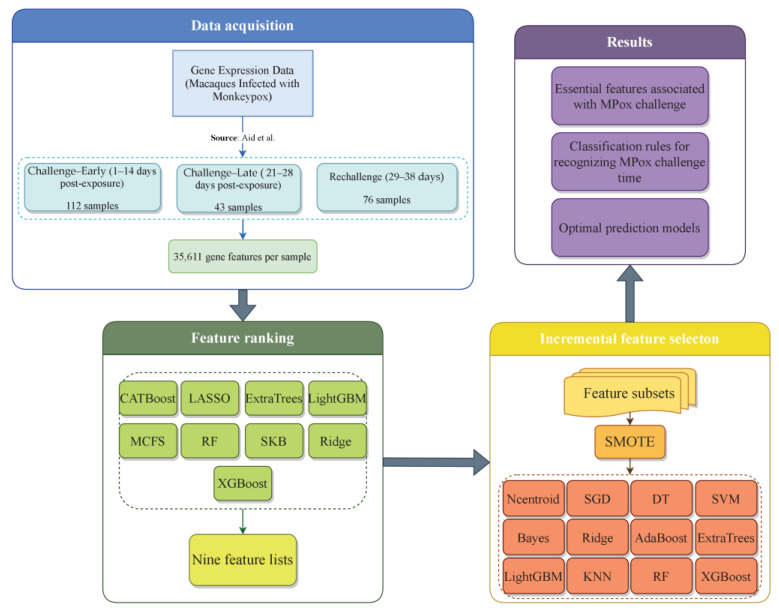
Workflow of Mpox infection time series gene expression data analysis for identifying key features associated with Mpox infection progression and control. We analyzed data on gene expression characteristics of Mpox infection in rhesus macaques at three distinct stages: early challenge (days 1–14), late challenge (days 21–28), and rechallenge (days 29–38). Employing nine feature ranking algorithms to analyze the data, nine feature lists were obtained. Then, these lists were fed into the IFS framework to extract essential features, constructing classification rules and optimal prediction models [30].

Figure 1 shows how this investigation was conducted. We analyzed time series data on Mpox infection in detail, which contained three phases: within two weeks after the first exposure (days 1–14), within four weeks after exposure (days 21–28), and during the rechallenge phase (days 29–38). Our workflow was mainly divided into four stages: data acquisition, feature ranking, IFS, and generating results. The gene expression data on different time points after infection with Mpox virus was downloaded from GEO. Then, feature significance assessment was rigorously performed with nine feature ranking algorithms, generating nine feature lists. The IFS method was followed to analyze each feature list, yielding essential features, optimal prediction models, and classification rules. This procedure incorporated SMOTE and twelve classification algorithms. The following sections detail the results obtained at each stage of the present study.

### 3.1. Feature Ranking Results of Features in Order of Importance

In this study, we utilized nine feature ranking algorithms to pinpoint key markers linked to Mpox. These algorithms included CATBoost, LASSO, ExtraTrees, LightGBM, MCFS, RF, SKB, Ridge, and XGBoost. Each algorithm produced a list comprising 35,611 features. Of them, SKB has 2000 features while the LightGBM feature list includes 33,527 features. Table S1 offers the nine feature lists. If features are assigned high ranks in one list, this suggests that they were important in one aspect. For convenience, these lists were called the CATBoost, LASSO, ExtraTrees, LightGBM, MCFS, RF, SKB, Ridge, and XGBoost feature lists.

### 3.2. IFS Results and Feature Intersections for Finding Key Features

**Figure 2:**
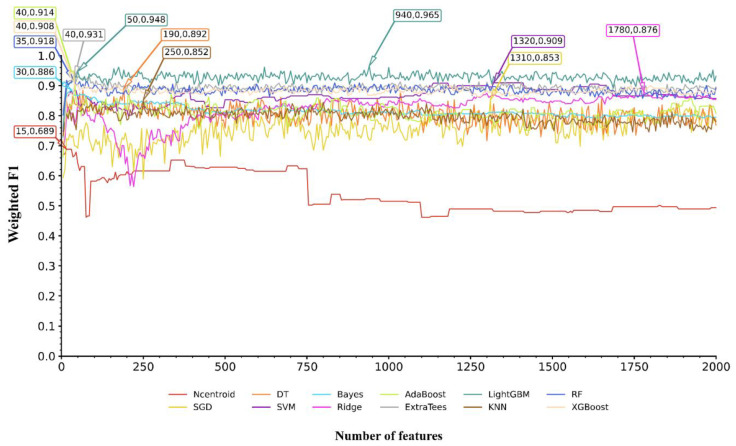
IFS curves for evaluating the performance of the 12 classification algorithms on the CATBoost feature list. The highest weighted F1 for each classification algorithm is marked on the curve, along with the number of features used. The full names of twelve classification algorithms can be found in Section Abbreviations.

From the nine feature lists, many features were included. If all possible feature subsets were considered in the IFS method, this would cost a large amount of time. On the other hand, not many genes should be associated with Mpox infection progression and control. Thus, it was not necessary to consider all features. Here, we only considered the top 2000 features in each list. To further accelerate the IFS procedures, we used the IFS method with a step size of five; i.e., each feature subset was constructed by adding the following five features in the list rather than the following one feature. Accordingly, 400 feature subsets were built from each feature list. In each feature subset, SMOTE was first adopted to balance the sizes of three classes, then twelve prediction models were built based on twelve classification algorithms (Ncentroid, SGD, DT, SVM, Bayes, Ridge, AdaBoost, ExtraTrees, LightGBM, KNN, RF, and XGBoost). Each model was evaluated by ten-fold cross-validation. The results were counted as ACC, MCC, macro F1, and weighted F1, which are available in Table S2 (each sheet corresponds to the IFS results from one feature list). To clearly show the performance of models with the same classification algorithm and different top features, an IFS curve was plotted using weighted F1 as the Y-axis and the number of features used as the X-axis for each classification algorithm. Figure 2 shows the IFS curves of the CATBoost feature list, whereas the IFS curves of the other eight feature lists are shown in Figures S1–S8.

The IFS results from the nine feature lists were analyzed in the same way. Here, we took the IFS results from the CATBoost feature list as an example. According to Figure 2, the highest weighted F1 values for the 12 classification algorithms were 0.689, 0.853, 0.892, 0.909, 0.886, 0.876, 0.914, 0.931, 0.965, 0.852, 0.918, and 0.908. This performance was obtained by using the top 15, 1310, 190, 1320, 30, 1780, 40, 40, 940, 250, 35, and 40 features in the CATBoost feature list. These features were termed as the optimal features for the classification algorithms. Accordingly, the optimal prediction model was built for each classification algorithm using its optimal features. Their detailed performance is listed in Table S3. Evidently, the optimal LightGBM model generated the best performance, with a weighted F1 of 0.965. This classifier was called the optimal model in the CATBoost feature list, and features used in this classifier (the top 940 features in the CATBoost feature list) were picked up as the optimal features extracted from the CATBoost feature list.

The same analysis was conducted on the IFS results of other feature lists. The optimal models using 12 classification algorithms on each feature list can be found, and their performance is provided, in Table S3. Then, the optimal model on each feature list was obtained. It was interesting that all optimal models used LightGBM as the classification algorithm. Their detailed performance and the number of features used are listed in Table 1. It can be found that the optimal model on the LightGBM feature list has evident advantages compared with the optimal classifiers on other feature lists. This model can be a latent useful tool to identify samples at different stages of Mpox infection.

**Figure 3:**
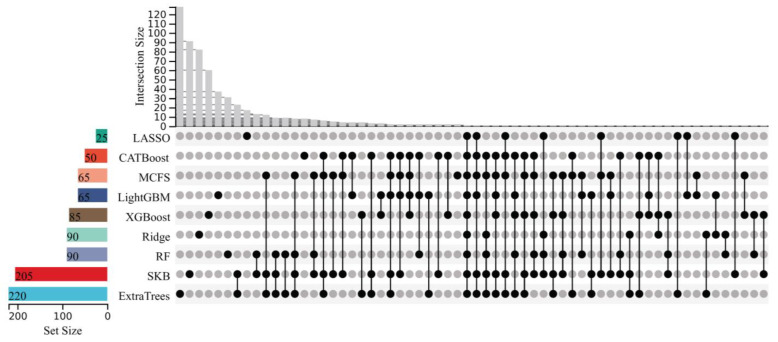
Upset graph of the feature subsets used for constructing the suboptimal prediction models on nine feature lists. “Set Size” is the count of the number of features in each set; “Intersection Size” is the count of the number of features after taking the intersection of some feature sets; the black dots indicate the unique features of a feature set; and the lines between the dots indicate the unique intersection of different feature sets.

With the above arguments, the optimal features on each feature list were identified, which can be used to construct the optimal prediction model. Clearly, they contributed to classifying samples into different stages of Mpox infection. Investigation of them is helpful to uncover the progression of Mpox infection. However, all optimal models needed many features (>200), which did not make detailed analysis easy. In view of this, we further extracted the most essential features. The IFS results from nine feature lists were checked again. The LightGBM model, using much fewer features but yielding similar performance to the optimal model, was identified on each feature list. Its performance and the numbers of features used are also listed in Table 1. These models used the top 50, 25, 220, 65, 65, 90, 205, 90, and 85 features in the corresponding lists. However, their performance was slightly lower than that of the optimal models. For example, the LightGBM model on the CATBoost feature list adopted the top 50 features and yielded a weighted F1 of 0.948. Compared with the corresponding information on the optimal model (940 features and weighted F1 of 0.965) on the same feature list, the features were much fewer in number, whereas the weighted F1 declined by 0.017. The above-obtained models on nine feature lists were called the suboptimal prediction models. Evidently, the features used in these models were more essential than others used in the optimal models. We picked up these features, comprising nine feature subsets. The overlap and relationships among these subsets are shown in detail in an upset graph, as illustrated in Figure 3. It can be found that several features belonged to multiple feature subsets. This meant that they were identified to be essential by multiple feature ranking algorithms, underscoring their potential relevance at distinct stages of Mpox virus infection. Table S4 lists the features exactly in one, two, or more feature subsets. Notably, one gene feature (CD19) was in exactly nine subsets, and three gene features (HS3ST1, SRPK3, and MS4A1) belonged to exactly six subsets. In this study, we mainly focused on gene features belonging to multiple subsets, as illustrated with representative examples in Section 4.1. Our research reveals important aspects of gene expression linked to the immune system’s response to Mpox virus infection over time. The temporal dynamics of gene expression and stage-specific immune features were investigated through classification rule-based analysis (Section 4.2), which provided representative examples associated with the early, late, and rechallenge phases. These findings highlight unique immunological patterns, particularly in secondary infections, and offer valuable insights into the evolving immune landscape during the course of Mpox virus infection.

### 3.3. Classification Rules Yielded by Decision Tree

The DT was also employed in the IFS method. Although the optimal DT model was inferior to the optimal LightGBM model on all feature lists, it has a special merit. As a white-box algorithm, its classification procedure can be observed, which is helpful for understanding its classification principles. Valuable insights on Mpox infection can be extracted from these principles. According to the optimal DT model on each feature list, we picked up its optimal features and employed all samples to train a new tree. From this tree, a rule group could be extracted, where each rule represented a path from the root to one leaf. All rule groups on the nine feature lists are available in Table S5, where each sheet corresponds to rules on one feature list. Each rule contained some conditions and one result (one of three classes). Each condition consisted of one gene feature and the threshold of its expression level. The combination of some conditions indicated a special expression pattern in the results, providing new materials for investigating the stages of Mpox infection. An extensive examination of the important genes in some rules and their significance for comprehending Mpox infection can be found in Section 4.

### 3.4. PPI Network Construction for Genes Identified in the Optimal Feature Sets

**Figure 4:**
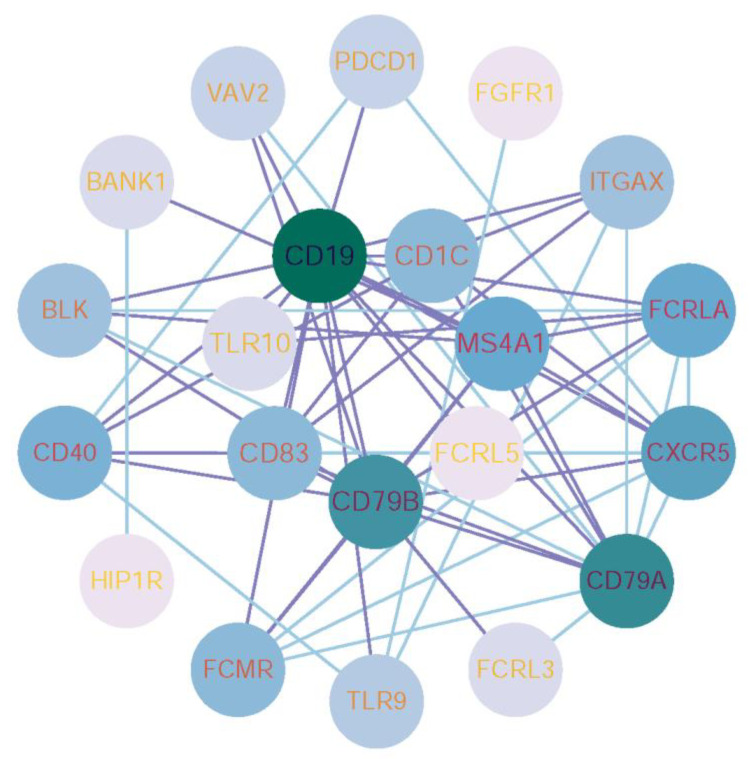
Protein-protein interaction (PPI) network visualization of key genes associated with Mpox challenge. The PPI network was constructed using STRING and key features from Table S4, visualized in Cytoscape. The network includes 21 nodes (e.g., CD19, CD79A, CXCR5), with node color and size representing centrality. Key interactions are highlighted in purple and positioned centrally.

As listed in Table S4, some genes belonged to multiple feature subsets. Here, we selected the genes belonging to more than three feature subsets for PPI network analysis. These results were imported into Cytoscape [60] for visual analysis and further screening of the PPI network, as shown in Figure 4. Interactions were determined by a CONFIDENCE score higher than 0.400, in order to highlight the main modules. For the visualization, only those scoring the highest were selected. From there, new clustering networks were created. This network contains 21 nodes, including CD19, CD79A, CD79B, CXCR5, and MS4A1, which are centrally positioned. Labels and nodes are colored according to their degree. Interactions of the key features discussed in Section 4.1 are highlighted with purple connecting lines, and these nodes are placed at the center.

### 3.5. Biological Functions of Key Genes Associated with MPox Challenge

**Figure 5:**
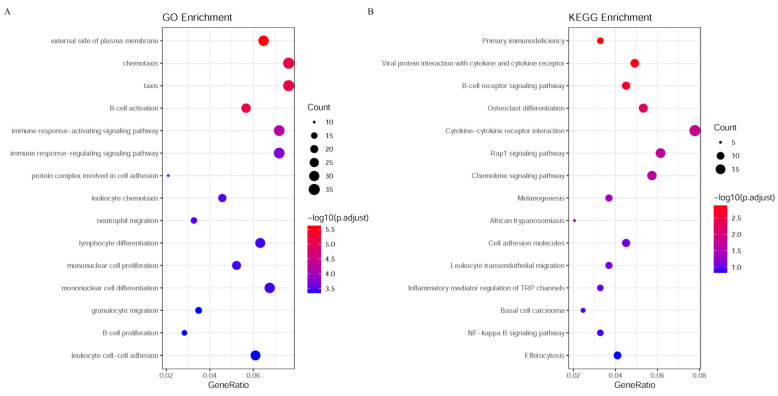
Enrichment analysis of GO and KEGG pathways. (**A**) The enrichment analysis results for GO; (**B**) The enrichment analysis results for KEGG pathways. The color gradient from blue to red indicates the range of adjusted *p*-values, with blue representing lower *p*-values (more significant enrichment) and red representing higher *p*-values (less significant enrichment). The size of the dots represents the number of genes involved in each biological process or pathway, with larger dots indicating a higher gene count.

For the gene features listed in Table S4, the enrichment analysis was conducted on them. The results revealed robust associations with Mpox challenge and signaling pathways. GO terms related to immune response, including “B cell activation,” “immune response−activating signaling pathway” and “immune response−regulating signaling pathway”, exhibited significant adjusted *p*-values and high gene counts (Figure 5A). Consistently, KEGG pathway analysis identified “Primary immunodeficiency” and “Viral protein interaction with cytokine and cytokine receptor” as the top enriched pathways (Figure 5B).

## 4. Discussion

The feature subsets at the intersection of different ranking algorithms, combined with the classification rules extracted using SMOTE and DT processing, bear critical importance for explaining the immune response at different stages of Mpox infection. To further support these findings, Table S6 summarizes the immune-related functions of representative genes identified across multiple feature subsets, based on curated annotations from publicly available databases such as GeneCards [63]. These annotations highlight the involvement of key genes in immunological processes including B-cell activation, antigen presentation, and innate immune signaling. We further analyzed several of these key genes, which helped us to understand how the expression pattern of specific genes corresponds to the rhesus macaques’ immune responses at different stages of infection, thereby enhancing our comprehension of disease course and host–pathogen interaction.

### 4.1. Integrative Analysis of Ranked Gene Features

CD19 was found to be a classification feature for classification by all nine algorithms. CD19 is a critical surface molecule on B cells; it serves as a co-receptor for BCR signaling in order to facilitate B-cell activation and antibody production. Through its interaction with BCR and other signaling molecules, CD19 amplifies the antigen recognition and responsiveness of B cells, thereby playing an important role in their activation, differentiation, and antibody production. It also represents an important immune checkpoint that helps in the control and maintenance of immune responses with repercussions for antibody production and the development of memory B cells during a viral infection [64]. CD19+ plasmablasts and Mpox A29-specific B cells increase progressively, paralleled by increased levels of anti-Mpox A29 antibodies after Mpox infection [65]. Due to the central position of B cells in humoral immunity, their role most probably involves quick differentiation into plasma cells, continuous production of antibodies, and formation of memory cells that ensure long-lasting antiviral immunity. Consequently, CD19 may serve as an important classification feature during the late challenge and rechallenge phases.

Genes such as MS4A1, CD83, FCRL5, and CD79B have been identified by numerous algorithms as characteristic genes with classification potential and are closely related to B-cell function. These genes are highly implicated in the regulation of B-cell activation, differentiation, and antibody production. MS4A1 is a gene encoding for a surface protein on B cells; its expression is noted in mature and memory B cells. It has implications in cell proliferation, differentiation, and calcium signaling and is commonly a target in antibody therapies [66]. CD83 is expressed on activated B cells, dendritic cells, and activated T cells, where it promotes B-cell activation and immune responses and has other immunoregulatory properties [67]. FCRL5 is highly expressed in memory B cells and modulates B-cell activity and antibody production [68,69]. CD79B is a part of the B-cell receptor complex and pairs with CD79A in the transmission of BCR signals, hence supporting B-cell proliferation, differentiation, and survival, making this an element indispensable in normal B-cell function [70]. B cells are the very key initiators in the humoral immune response, which start their action by recognizing and binding viral antigens via their surface antibodies. This leads to the production of specific antibodies that neutralize the virus and prevent its spreading. Immune responses induced by Mpox infection and vaccination studies have demonstrated that activated B cells can further differentiate into short-lived, antibody-secreting plasmablasts [71]. This B-cell response occurs simultaneously with the beginning of infection, permitting the generation of antibodies, which is of great importance for containing viral replication and spread. Later, during infection, B cells are in charge of immune memory formation [2]. This was confirmed by studies of peculiarities in level changes in a specific antibody after Mpox virus infection [72]. Taken together, our results strongly support that B cells are major players in the immunological response to Mpox infection and show extensive functional changes due to the infection.

The TLR10 gene has been predicted as an important feature by four different algorithms. The TLR10 gene encodes a protein that belongs to a family of proteins called Toll-like receptors (TLRs), which play a critical role in pathogen recognition and the activation of the innate immune response. TLRs are receptors located both on the cell surface and within endosomes that facilitate the detection of pathogen-associated molecular patterns and damage-associated molecular patterns [73]. The active TLRs can induce the signaling pathways of MAPKs and NF-κB, leading to the production of pro-inflammatory cytokines and chemokines [74]. Studies show that TLRs play an important role in Mpox infection in recognizing viral nucleic acid, amplifying the inflammatory response, and counteracting viral replication [75]. They can activate CD11 cells and dendritic cells to upregulate genes encoding pro-inflammatory cytokines like IL-6, TNF-α, IL-1β, and IL-12 [12,76]. In addition, TLRs are involved in the recruitment of immune cells and differentiation into memory cells after infection [75].

Further, we identified a number of genes involved in the MHC, including MAMU.DOA and CD1C. MAMU.DOA is an MHC class II gene in rhesus macaques whose product takes part in the antigen presentation process, thus helping the immune system to recognize pathogens and induce appropriate responses. The non-classical MHC molecule CD1C is mainly expressed in dendritic cells and B cells, presenting lipid and glycolipid antigens to T cells, thereby activating specific immune responses [77]. Mamu molecules have been implicated in both the restriction and the recognition of T-cell epitopes [78]. The expression of MHC II molecules is mainly confined to antigen-presenting cells, including dendritic cells, macrophages, and B cells. They bind with viral antigens and present them to CD4+ helper T cells, which start a cell-mediated immune response [79]. Studies have shown that MHC epitope prediction and antigen affinity analysis are valuable for developing appropriate treatments for Mpox and facilitating the design of effective Mpox vaccines [80]. Accordingly, the expression of MHC molecules may be increased after viral infection, which is important for recognition and the induction of specific immune responses against the virus. Hence, genes corresponding to MHC molecules whose expression is relatively low in the early challenge phase can be used as important features for classification.

**Figure 6:**
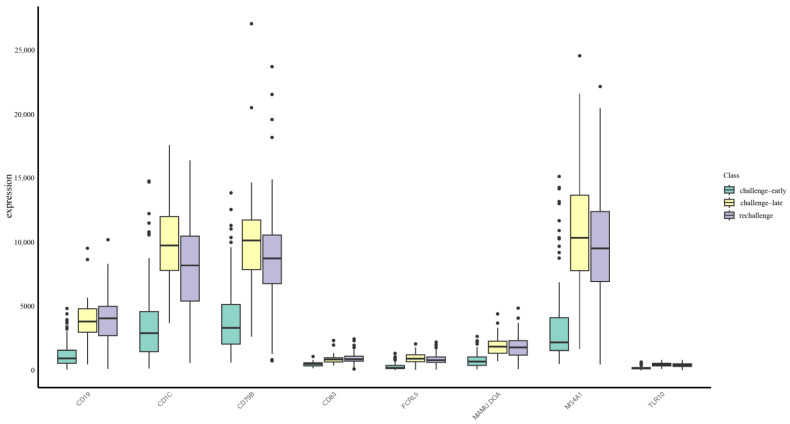
Gene expression levels in three Mpox infection stages. The *X*-axis represents selected genes, and the *Y*-axis shows their expression levels. Expression levels for each gene are categorized into three groups (indicated by different fill colors): challenge-early (days 1–14), challenge-late (days 21–28), and rechallenge (days 29–38). Each boxplot displays the median, interquartile range, and potential outliers.

These results reflect that immune-related genes like CD19, MS4A1, CD83, FCRL5, CD79B, TLR10, MAMU.DOA, and CD1C are important in the immune response against Mpox infection. We visualized the expression level distribution of these features; Figure 6 represents that most of the genes are relatively highly expressed during the late challenge and rechallenge phases, consistent with previous studies [65,71,72,81,82]. Of these, the most important feature is represented by CD19, which was identified as a classification feature by all the algorithms, and was a central node in the PPI network (Figure 4). The genes encoding for CD19 and other B-cell genes, participating in the processes of initiation and maintenance of humoral immunity, resulting in early neutralization of viruses and providing long-term immune memory, are important. The TLRs, such as TLR10, enhance recognition of viral nucleic acids, with increased inflammatory response, thus contributing to control over viral replication. MHC-related genes, like MAMU.DOA and CD1C, involved in antigen presentation and T-cell activation, are important key molecules of pathogen recognition and immune response. This data may help in further deducing some important aspects of the immunological mechanisms involved in Mpox infection and may be useful in targeting therapies or developing an effective vaccine.

### 4.2. Analysis of Features Within Classification Rules

We further examined the expression of the genes mentioned in the previous section, as well as other potentially significant genes, in relation to the three different infection stages within the classification rules results. In the classification rules derived from the LASSO algorithm, we observed that relatively low expression of CD19 was identified as a characteristic of the early challenge stage. This may be in relation to the production of antibodies by B cells at a late challenge stage and rapid activation of memory immune responses at the rechallenge stage. This observation agrees with dynamic descriptions of immune cell activity across these stages, as reported in our data sources, and supports the reliability of our findings. Additionally, within these classification rules, high expression of the gene SLAMF6 was associated with the rechallenge stage, while lower expression was linked to the late challenge stage. In contrast, the classification rules from the Ridge algorithm identified IL2RG as an important gene related to the rechallenge stage.

SLAMF6 is an immune receptor involved in the activation and functional regulation of T cells, NK cells, etc., facilitating cooperative interactions and effective immune responses during antiviral defense [83]. IL2RG, the common γ-chain shared by various cytokine receptor complexes, plays a role in the growth and activation of T cells and NK cells and contributes to humoral immunity. Previous studies have shown that SLAMF6 expression is associated with improved progression-free and overall survival in breast cancer and melanoma, in which SLAMF6+ CD8+ T cells maintain the ability of multifunctionality and contribute to long-term tumor control [84]. We therefore postulate that the high expression of SLAMF6 in Mpox rechallenge may be related to the quick response and activation of memory T cells, resulting in a prompt initiation of immune responses. IL2RG has been shown to contribute to the immune response during secondary viral infections by promoting the swift entry of memory cells into the cell cycle and enabling IL-2 signal-dependent competitive proliferation [85]. Interfering with cytokine signaling by blocking anti-IL2RG antibodies can reduce T-cell activation and proliferation, especially affecting effector memory T cells [86]. We believe that in reinfection, the highly expressed IL2RG might support the proliferation and function of memory T cells and NK cells to rapidly recognize and clear the virus from the body.

In addition to the well-established immune-related genes, our analysis identified a set of previously unreported candidates—such as HS3ST1, SPAG16, MTARC2, F2RL1, and ZBTB32—implicated across multiple feature subsets, suggesting potentially overlooked roles in Mpox-related immune responses. Notably, heparan sulfate-glucosamine 3-sulfotransferase 1 (HS3ST1) emerged as a critical feature in the classification rules identified by the majority of feature ranking algorithms. Its expression was consistently lower during the early infection phase but markedly upregulated during the late and rechallenge phases, implying a potential role in shaping adaptive immunity. As a sulfotransferase involved in the modification of heparan sulfate, HS3ST1 may influence viral entry, inflammatory signaling, or immune cell trafficking [87]. Moreover, sperm-associated antigen 16 (SPAG16) was identified in SKB-derived classification rules as being highly expressed during the early challenge phase. Although traditionally associated with ciliary structure, SPAG16 has also been recognized as a target of humoral immune responses in multiple sclerosis (MS) [88]. Our findings suggest that SPAG16 may contribute to early immune signaling during Mpox infection, potentially modulating the activation of B cells or other immune cell populations. This modulation could enhance antibody production and facilitate an effective initial defense against viral invasion. Similarly, mitochondrial amidoxime reducing component 2 (MTARC2) was highlighted in the rules on the LightGBM feature list, showing elevated expression at the early challenge stage of infection. MTARC2 is implicated in nitric oxide synthesis and metabolic regulation, and given the pivotal role of nitric oxide in antiviral defense and immune modulation [89,90], we propose that its high expression may reflect a characteristic metabolic or immune activation signature in the early phase of Mpox infection.

In summary, our findings expand the current understanding of host immune responses to Mpox by identifying both well-established and underexplored genes—such as HS3ST1, SPAG16, and MTARC2—that may serve as novel targets for mechanistic investigation and therapeutic intervention. Moreover, our study provides a systematic framework for dissecting dynamic immune responses to Mpox infection through the integration of time series transcriptomic data and ML techniques. The identified key genes—such as CD19, MS4A1, and TLR10—not only offer insight into B-cell activation, innate immunity, and antigen presentation but also suggest potential applications in disease staging, therapeutic target discovery, and rational vaccine design. However, limitations exist. The reliance on rhesus macaque models introduces interspecies variation, which may limit direct translational applicability to humans. Additionally, the performance and interpretability of ML models are influenced by data quality and algorithm selection. Future validation in human clinical samples and functional studies of candidate genes is needed to substantiate their clinical relevance. Overall, our findings enhance the mechanistic understanding of host–pathogen interactions in Mpox and lay a foundation for developing diagnostic and therapeutic strategies.

## 5. Conclusions

In order to uncover important immune response aspects, this study designed a ML-based analysis framework and applied it to macaque time series data collected after Mpox infection. Several essential genes were identified to have strong associations with Mpox infection by multiple feature ranking methods. Among them, CD19, MS4A1, and TLR10 have been found to be essential for B-cell activation, antibody generation, and the innate immune response, whereas some novel key genes (HS3ST1, SPAG16, and MTARC2) provided new directions for further investigating Mpox infection, such as validation through additional experiments or animal studies. Furthermore, an efficient prediction model was constructed, which can be a latent tool to identify samples at different stages of Mpox infection.

## Figures and Tables

**Table 1:** Performance of the optimal and suboptimal prediction models on nine feature lists.

Feature List	Classification Algorithm	Number of Features	ACC	MCC	Macro F1	Weighted F1
CATBoost feature list	LightGBM ^$^	940	0.965	0.945	0.965	0.965
	LightGBM ^#^	50	0.948	0.916	0.948	0.948
LASSO feature list	LightGBM ^$^	1045	0.931	0.890	0.920	0.932
	LightGBM ^#^	25	0.900	0.842	0.884	0.902
ExtraTrees feature list	LightGBM ^$^	1155	0.944	0.911	0.936	0.944
	LightGBM ^#^	220	0.922	0.876	0.918	0.922
LightGBM feature list	LightGBM ^$^	200	0.991	0.986	0.991	0.991
	LightGBM ^#^	65	0.970	0.952	0.964	0.970
MCFS feature list	LightGBM ^$^	1900	0.952	0.925	0.947	0.953
	LightGBM ^#^	65	0.931	0.889	0.923	0.931
RF feature list	LightGBM ^$^	1230	0.944	0.911	0.941	0.944
	LightGBM ^#^	90	0.900	0.841	0.891	0.901
SKB feature list	LightGBM ^$^	1565	0.935	0.899	0.931	0.936
	LightGBM ^#^	205	0.879	0.805	0.866	0.879
Ridge feature list	LightGBM ^$^	860	0.944	0.911	0.941	0.944
	LightGBM ^#^	90	0.892	0.830	0.882	0.893
XGBoost feature list	LightGBM ^$^	255	0.974	0.959	0.972	0.974
	LightGBM ^#^	85	0.944	0.910	0.941	0.944

^$^: Optimal prediction model; ^#^:suboptimal prediction model.

## Data Availability

Data is contained within the article or Supplementary Materials.

## Supplementary Materials

The following supporting information can be downloaded at https://www.mdpi.com/article/10.3390/life15071039/s1, File S1: Description of some machine learning algorithms; Table S1: Feature ranking results obtained using CATBoost, LASSO, ExtraTrees, LightGBM, MCFS, RF, SKB, Ridge, and XGBoost; Table S2: Performance of IFS with 12 different classification algorithms on CATBoost, LASSO, ExtraTrees, LightGBM, MCFS, RF, SKB, Ridge, and XGBoost feature lists; Table S3: Performance of the optimal prediction models based on 12 classification algorithms on nine feature lists; Table S4: Intersection of nine essential feature sets extracted from the CATBoost, LASSO, ExtraTrees, LightGBM, MCFS, RF, SKB, Ridge, and XGBoost feature lists; Table S5: Classification rules generated by decision trees using their optimal features on nine feature lists; Table S6: Immune-related functions of representative genes identified across multiple feature subsets; Figure S1: IFS curves for evaluating the performance of the 12 classification algorithms on the LASSO feature list; Figure S2: IFS curves for evaluating the performance of the 12 classification algorithms on the ExtraTrees feature list; Figure S3: IFS curves for evaluating the performance of the 12 classification algorithms on the LightGBM feature list; Figure S4: IFS curves for evaluating the performance of the 12 classification algorithms on the MCFS feature list; Figure S5: IFS curves for evaluating the performance of the 12 classification algorithms on the RF feature list; Figure S6: IFS curves for evaluating the performance of the 12 classification algorithms on the SKB feature list; Figure S7: IFS curves for evaluating the performance of the 12 classification algorithms on the Ridge feature list; Figure S8: IFS curves for evaluating the performance of the 12 classification algorithms on the XGBoost feature list. Reference [91] is cited in the Supplementary Materials.

## Abbreviations

The following abbreviations are used in this manuscript:

Mpox	Monkeypox virus
IFS	Incremental feature selection
CATBoost	Categorical Boosting
LASSO	Least Absolute Shrinkage and Selection Operator
ExtraTrees	Extremely Randomized Trees
LightGBM	Light Gradient Boosting Machine
MCFS	Monte Carlo Feature Selection
RF	Random Forest
SKB	SelectKBest
XGBoost	eXtreme Gradient Boosting
SMOTE	Synthetic Minority Oversampling Technique
Ncentroid	Nearest Centroid Classifier
SGD	Stochastic Gradient Descent
DT	Decision Tree
SVM	Support Vector Machine
Bayes	Naïve Bayes Classifier
AdaBoost	Adaptive Boosting
KNN	K-Nearest Neighbors
ML	Machine learning
ACC	Accuracy
MCC	Matthews correlation coefficient
PPI	Protein–protein interaction
GO	Gene ontology
BP	Biological process
MF	Molecular function
CC	Cellular component
KEGG	Kyoto Encyclopedia of Genes and Genomes
TLR	Toll-like receptor
HS3ST1	Heparan sulfate-glucosamine 3-sulfotransferase 1
SPAG16	Sperm-associated antigen 16
MTARC2	Mitochondrial amidoxime reducing component 2
