# Supplementary material for: Unveiling Immune Response Mechanisms in Mpox Infection Through Machine Learning Analysis of Time Series Gene Expression Data
Source: Life (Basel). 2025 Jun 30;15(7):1039. doi: 10.3390/life15071039 (PMC12301010; doi:10.3390/life15071039)
Supplement: Supplementary file 1 [file life-15-01039-s001.zip › File S1.pdf]

File S1. Description of some machine learning algorithms.

1. Feature ranking methods

1.1 Categorical Boosting

CATBoost is a state-of-the-art open-source gradient boosting library developed by Yandex [1]. It stands out in the field of machine learning for its ability to efficiently handle categorical variables directly, without the need for extensive preprocessing that is typically required by other algorithms. It employs Ordered Boosting which is a modification of the traditional gradient boosting method. It helps in fighting overfitting by preventing the same data samples from being used in both the construction of the trees and their subsequent evaluation. Feature importance in CATBoost is often estimated by Prediction Values Change. This method measures the impact of a feature on the accuracy of the model. It is calculated by removing or shuffling the feature's values and observing the change in the model's prediction accuracy. A significant change suggests that the model heavily relies on that feature.

1.2 Least Absolute Shrinkage and Selection Operator

LASSO is a regression analysis method that both performs variable selection and regularization in order to enhance the predictive power and interpretability of the generated statistical model. Another plus point of LASSO is that it can estimate the relative contributions of different features in a dataset [2]. The LASSO technique encourages the sum of absolute values of regression coefficients to be less than a fixed value, which causes some coefficients to shrink to zero. The greater the penalty λ, the more the coefficients shrink exactly to zero. The magnitude of a coefficient that does not shrink to zero can be interpreted as an indication of the importance of the corresponding feature, that is, a larger absolute value indicates higher importance.

1.3 Extremely Randomized Trees

ExtraTrees, the abbreviation of Extremely Randomized Tree, is an ensemble machine learning algorithm similar to random forest, aiming at solving some shortcomings of traditional random forest methods [3]. Like random forest, ExtraTrees constructs multiple decision trees during training and aggregates their predictions to improve generalization ability and robustness. However, it introduces even more randomness in the way splits are computed, which often leads to increased model performance, especially in terms of variance reduction. ExtraTrees uses random thresholds for each feature rather than searching for the best possible thresholds (as in random forests). This randomness is applicable not only to the selection of features, but also to the way of splits within the tree. Unlike random forest, where each tree is constructed by bootstrapping, ExtraTrees usually use the whole original data set to grow each tree.

1.4 Light Gradient Boosting Machine

LightGBM is an advanced implementation of the gradient boosting algorithm [4]. Developed by Microsoft, it's designed to be more efficient and scalable than traditional gradient boosting methods like XGBoost, particularly when dealing with large-scale and high-dimensional data. LightGBM is popular because of its efficiency and effectiveness in a wide range of predictive tasks. Different from the traditional gradient boosting technique which uses all features to determine the best splits, LightGBM uses Exclusive Feature Bundling method, which bundles the mutually exclusive features together to reduce the dimension of the data. This speeds up the training process and reduces memory usage.

The importance of features can be estimated by the gain method. This method measures the accuracy improvement or information gain brought by the feature. The information gain is the total reduction of loss provided by splitting nodes on the feature, which is aggregated over all trees. A higher value of the metric means that the feature is more important.

1.5 Monte Carlo Feature Selection

MCFS is a powerful statistical method to determine the importance of each feature in large and complex data sets, in which the number of features may exceed the number of samples [5]. MCFS uses stochastic process to evaluate the importance of features over multiple iterations where random data sets are constructed. The process can be summarized into the following steps: 1. MCFS randomly selects feature subsets from the whole feature set. 2. For each feature subset, it creates many subsets of training data randomly. This dual-layer randomness increases the robustness of the feature importance assessment since features are tested using many segments of data. 3. For each data subset, build a decision tree. 4. Then, the importance of features is calculated according to several criteria: the weighted accuracy of each decision tree, the improvement of prediction accuracy brought by splits based on the feature, and the coverage of all splits based on the feature.

1.6 Random Forest

RF is a powerful ensemble learning method. It is based on the simplicity of decision trees, and enhances their accuracy and robustness by creating a forest of trees instead of relying on a single decision tree [6]. Random Forest applies the general technique of bootstrap aggregation or bagging to create training data subsets. Each tree is trained on a random sample of data in the training set, selected with replacement, which is called bootstrap sampling. When constructing each tree, the random forest randomly selects a subset of features at each split decision, instead of using all available features. Every tree in the forest is built as big as possible (a fully grown tree) without pruning. For the classification task, the random forest takes the class that gets the most votes from all trees as the output of the prediction.

The most common method used by random forest to calculate feature importance is based on the reduction of node impurity (for example, Gini impurity for classification, mean square error for regression), which results from splitting each node on a feature. Essentially, the more impurities a feature reduces, the more important it is. The impurity reductions of each feature over all trees in the forest are averaged to get the final importance score for the feature.

1.7 SelectKBest

SKB works by applying a statistical test to each feature individually to determine its importance relative to the response variable [7]. The method then retains the top K features that have the highest scores from these tests, effectively reducing the number of variables in the dataset. The choice of the statistical test depends on the type of data (categorical or continuous) and the problem at hand (classification or regression). Common tests for classification include Chi-squared test, ANOVA F-test and mutual information. Each feature is scored based on the selected statistical test. The score typically reflects the degree of independence between the feature and the target variable. Higher scores indicate a feature is more relevant to the target. The user specifies K, the number of top features to select. SelectKBest then retains these K features with the highest scores, and discards the rest.

1.8 Ridge Regression

Ridge regression, or also known as Tikhonov regularization, is a technique used in regression analysis to cope with multicollinearity among predictor variables [8]. By adding a penalty term to the least squares objective, ridge regression shrinks the regression coefficients, thus stabilizing the solution and reducing the variance of the estimates. This method enhances the generalization capabilities of regression models by imposing a penalty on the size of the coefficients. Ridge Regression can still perform well where ordinary least squares regression would fail due to multicollinearity (high correlations between predictor variables). Unlike some other regularization methods, ridge regression will not reduce any coefficient to zero. Therefore, it includes all the features in the model, which may be important when all the features are contributing.
After shrinkage, the magnitudes of the coefficients can provide insight into the relative importance of each feature. A larger (absolute) coefficient after regularization implicates that the corresponding feature plays a more important role in predicting the target variables. It is very important to standardize features in ridge regression before applying the model. Standardized features can ensure that regularization is uniformly applied to all features, thus making the coefficient magnitudes more comparable and interpretable.

1.9 eXtreme Gradient Boosting

XGBoost is an efficient and flexible gradient boosting library, which is popular among data scientists and machine learning practitioners [9]. Developed by Chen Tianqi, XGBoost is specially designed to be both computationally efficient and effective in a wide range of machine learning tasks. XGBoost introduces regularization term into the loss function, which helps to control over-fitting and makes it better than traditional gradient boosting. This is one of the key enhancements of XGBoost, compared with other boosting techniques.

Feature importance in XGBoost can be estimated by the Cover Method. This measures the relative quantity of observations concerned by a feature. It is defined as the number of times a feature is used to split the data weighted by the number of training data points that go through those splits. It reflects the structural importance of a feature in terms of the number of data points it impacts.

2. Classification algorithms

2.1 Nearest Centroid Classifier

The Nearest Centroid Classifier is a straightforward yet effective classification algorithm, part of the family of "centroid-based" models [10]. It is similar in spirit to the KNN but simplifies the classification task by representing each class by the central point of its members. The Nearest Centroid Classifier works as follows: 1. Centroid Calculation: First, for each class in the training dataset, the centroid is computed by taking the average of all instances (feature vectors) that belong to this class; this average vector defines the "center" of the class in the feature space. 2. Class Assignment: To classify a new instance, calculate the distance from the instance to the centroid of each category, and assign the instance to the class whose centroid is nearest to it. Euclidean distance is ordinarily used to measure distance, although other measures can also be used according to data characteristics.

2.2 Stochastic Gradient Descent

SGD is a widely used optimization algorithm in machine learning, particularly in large-scale learning tasks [11]. The SGD classifier employs optimization technique to fit linear models. It is especially effective for problems with a large number of samples

and features, where other optimization methods might be computationally expensive. The following is a brief description of how SGD works: 1. Starting from the initial values of model parameters (weights). 2. For each training example, calculate the gradient of the loss function (it measures the error between the predicted output and the actual output). 3. Take a small step (learning rate) in the opposite direction of the gradient, and adjust the model's parameters to decrease the loss. 4. Repeat the above steps for every instance in the training set, probably several times for the whole dataset (every pass over the whole dataset is referred to as an epoch).

2.3 Decision Tree

DTs are organized as tree-like models of decisions, where each internal node represents a "test" on an attribute; every branch represents the outcome of the test, and every leaf node represents a class label or a continuous value in the case of regression [12]. The process of building a decision tree involves recursively dividing data into subsets based on an attribute value that gets the most significant information gain (or the greatest uncertainty reduction) for classification tasks or the least mean square error for regression. Popular algorithms for building decision trees include ID3, C4.5 and CART. Decision tree is the basic component of many advanced machine learning models, and plays a significant role in many applications including strategic planning, medical care, finance and manufacturing.

2.4 Support Vector Machine

SVM is a powerful supervised machine learning algorithm [13]. SVM is highly praised for its ability to deal with high-dimensional data and its effectiveness in cases when the feature dimension exceeds the number of samples. SVM works by finding the hyperplane that best separates different classes in the feature space. In two dimensions, a hyperplane can be considered as a line separating two classes. In higher dimensions, it is a plane or hypersurface. SVM finds the hyperplane that has the maximum margin. A margin is defined as the distance between the hyperplane and the closest training samples from either class, known as support vectors. A larger margin means that the generalization error of the classifier is lower. For nonlinear separable data, SVM uses a technique called kernel trick. This involves transforming data into a higher dimensional space, in which a linear hyperplane can be used to divide data. In order to deal with overlapping classes (not completely separable), SVM introduces a regularization parameter, which controls the trade-off between realizing low error and minimizing model complexity to achieve better generalization.

2.5 Naïve Bayes Classifier

Bayes is a probabilistic machine learning model, which is widely used in classification tasks [14]. The core of Naïve Bayesian classifier is to apply Bayes' Theorem, which describes the probability of events based on prior knowledge of conditions that may be related to events. The model assumes that the features are independent given the class label, thus simplifying the calculation. This means that the existence of one attribute will not affect the existence of another attribute in the class context. Let $P(x_i | C_k)$ be likelihood, which is the probability of predictor x_i given class C_k, and $P(x_i)$ is the prior probability of the predictor. The Maximum Likelihood Estimation (MLE) is used to estimate the parameters of $P(x_i | C_k)$ from the training data. In order to predict the new observations, the classifier calculates the posterior probability of each class and selects the class with the highest probability.

2.6 Adaptive Boosting

AdaBoost works by training a sequence of weak classifiers (usually simple

decision trees, also known as decision stumps) on repeatedly modified versions of the data [15]. The predictions from all of them are then combined through a weighted majority vote to produce the final prediction. The fundamental steps involved in the AdaBoost algorithm are: 1. Initialize Weights: Each instance in the training dataset is initially assigned the same weight, which indicates the importance of the instance in the learning process. 2. A weak classifier is trained on the data, with the training process being influenced by the weights of the instances. 3. The classifier's accuracy is evaluated based on the weighted training data. 4. Instances that are incorrectly classified are given increased weights, whereas weights are decreased for those that are correctly classified. 5. Steps 2-4 are repeated for a predefined number of iterations. 6. Combine the Weak Classifiers: After training, each classifier is assigned a coefficient related to its accuracy, and these coefficients are used to weight the classifiers' votes in the final decision.

2.7 K-Nearest Neighbors

KNN algorithm is a simple, yet powerful machine learning method used for both classification and regression tasks [16]. It is based on the intuitive idea of predicting the properties of a given data point based on the properties of its nearest neighbors. Here is how KNN works: 1. Determine the number K. First, choose the number of neighbors, K. This is a critical parameter and can be selected based on cross-validation [17]. 2. Calculate Distance: For a given data point, calculate the distance (usually Euclidean) from this point to all other points in the training dataset. 3. Identify Nearest Neighbors: Identify the K closest training data points – the "nearest neighbors". 4. Vote for Labels: For classification, the algorithm assigns the class label based on a majority vote of these K nearest neighbors.

References

1. Dorogush, A.V.; Ershov, V.; Gulin, A. Catboost: Gradient boosting with categorical features support. *arXiv preprint arXiv:1810.11363* **2018**.
2. Tibshirani, R. Regression shrinkage and selection via the lasso. *Journal of the Royal Statistical Society: Series B (Methodological)* **1996**, *58*, 267-288.
3. Chen, T.; Guestrin, C. In *Xgboost: A scalable tree boosting system*, The 22nd ACM SIGKDD International Conference on Knowledge Discovery and Data Mining, 2016; Association for Computing Machinery: 2016; pp 785-794.
4. Ke, G.; Meng, Q.; Finley, T.; Wang, T.; Chen, W.; Ma, W.; Ye, Q.; Liu, T.-Y. Lightgbm: A highly efficient gradient boosting decision tree. *Advances in neural information processing systems* **2017**, *30*, 3146-3154.
5. Draminski, M.; Rada-Iglesias, A.; Enroth, S.; Wadelius, C.; Koronacki, J.; Komorowski, J. Monte carlo feature selection for supervised classification. *Bioinformatics* **2008**, *24*, 110-117.
6. Breiman, L. Random forests. *Machine learning* **2001**, *45*, 5-32.
7. Ayyanar, M.; Jeganathan, S.; Parthasarathy, S.; Jayaraman, V.; Lakshminarayanan, A.R. In *Predicting the cardiac diseases using selectkbest method equipped light gradient boosting machine*, 2022 6th International conference on trends in electronics and informatics (ICOEI), 2022; IEEE: pp 117-122.
8. Hoerl, A.E.; Kennard, R.W. Ridge regression: Biased estimation for nonorthogonal problems. *Technometrics* **1970**, *12*, 55-67.
9. Chawla, N.V.; Bowyer, K.W.; Hall, L.O.; Kegelmeyer, W.P. Smote: Synthetic minority over-

sampling technique. *Journal of Artificial Intelligence Research* **2002**, *16*, 321-357.

10. Levner, I. Feature selection and nearest centroid classification for protein mass spectrometry. *BMC bioinformatics* **2005**, *6*, 68.
11. Osho, O.; Hong, S. An overview: Stochastic gradient descent classifier, linear discriminant analysis, deep learning and naive bayes classifier approaches to network intrusion detection. *International Journal of Engineering and Technical Research* **2021**, *10*, 294-308.
12. Safavian, S.R.; Landgrebe, D. A survey of decision tree classifier methodology. *IEEE transactions on systems, man, and cybernetics* **1991**, *21*, 660-674.
13. Cortes, C.; Vapnik, V. Support-vector networks. *Machine Learning* **1995**, *20*, 273-297.
14. Rish, I. In *An empirical study of the naive bayes classifier*, IJCAI 2001 workshop on empirical methods in artificial intelligence, 2001; pp 41-46.
15. Freund, Y.; Schapire, R.E. A decision-theoretic generalization of on-line learning and an application to boosting. *Journal of computer and system sciences* **1997**, *55*, 119-139.
16. Cover, T.; Hart, P. Nearest neighbor pattern classification. *IEEE Transactions on Information Theory* **1967**, *13*, 21-27.
17. Kohavi, R. A study of cross-validation and bootstrap for accuracy estimation and model selection. In *Proceedings of the 14th international joint conference on Artificial intelligence - Volume 2*, Morgan Kaufmann Publishers Inc.: Montreal, Quebec, Canada, 1995; pp 1137–1143.
