# Supplementary figures and images for: Unveiling Immune Response Mechanisms in Mpox Infection Through Machine Learning Analysis of Time Series Gene Expression Data
Source: Life (Basel). 2025 Jun 30;15(7):1039. doi: 10.3390/life15071039 (PMC12301010; doi:10.3390/life15071039)

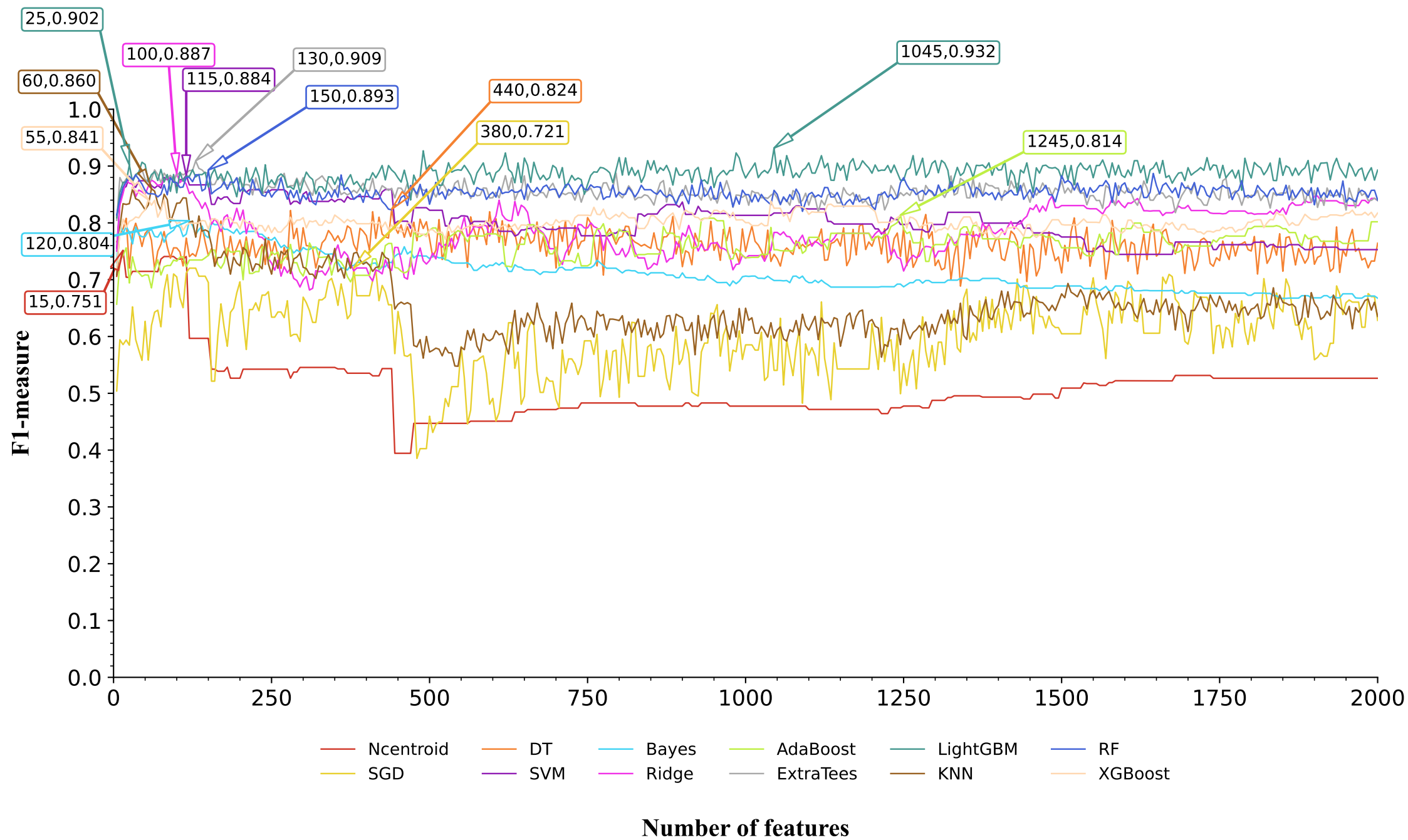

Supplement: Supplementary file 1 [file life-15-01039-s001.zip › Figure S1.pdf]

F1-measure

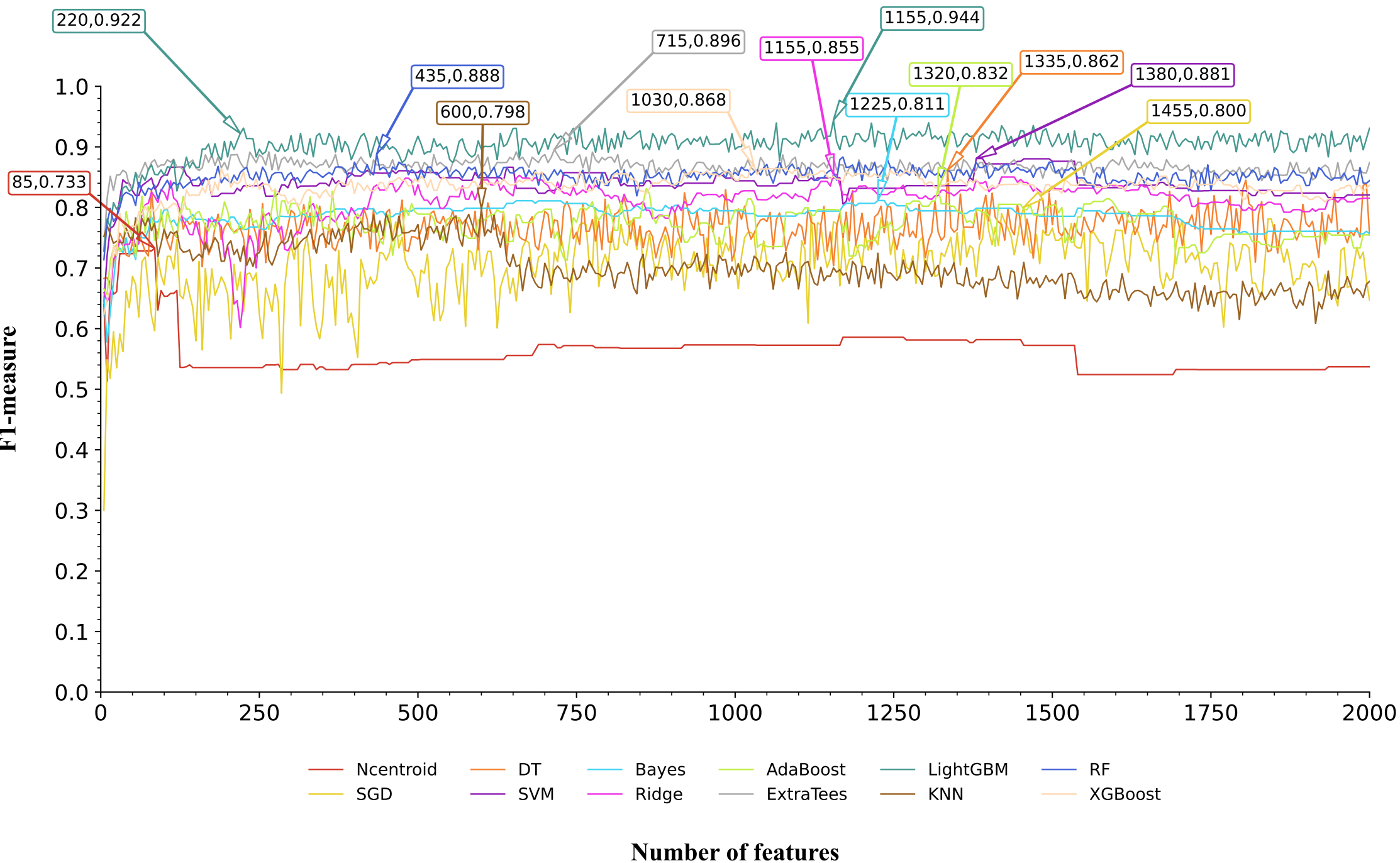

Supplement: Supplementary file 1 [file life-15-01039-s001.zip › Figure S2.pdf]

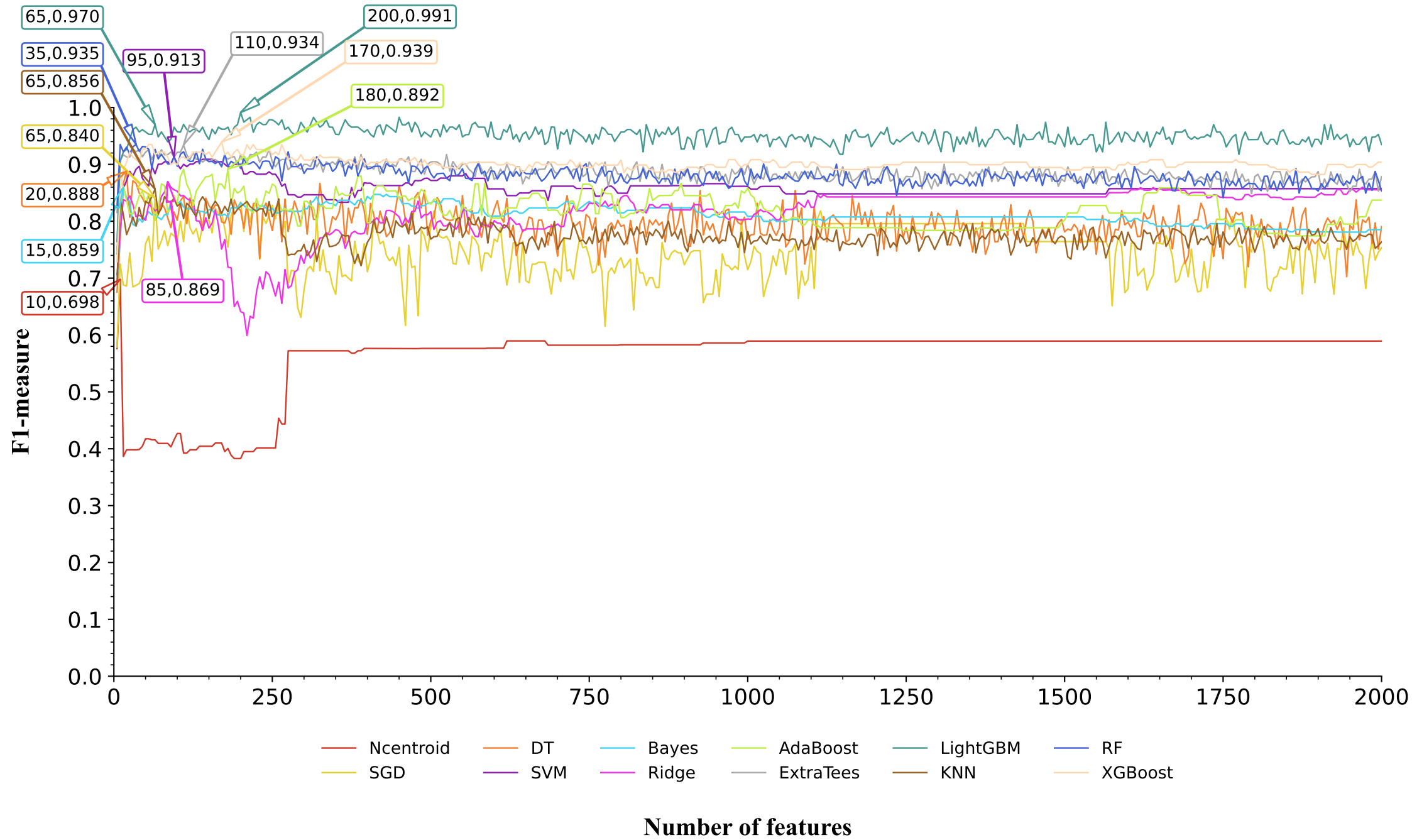

Supplement: Supplementary file 1 [file life-15-01039-s001.zip › Figure S3.pdf]

**F1-measure**

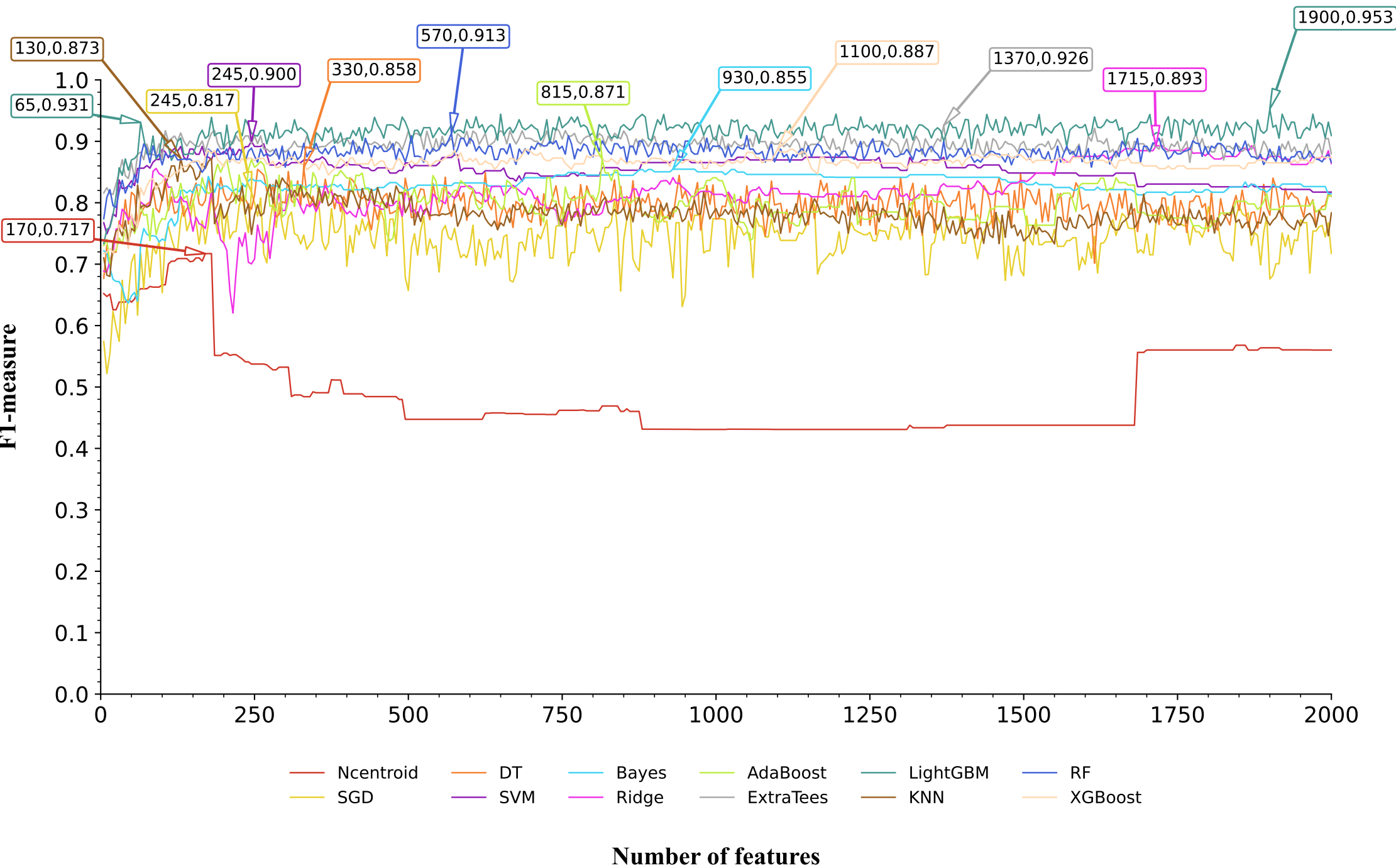

Supplement: Supplementary file 1 [file life-15-01039-s001.zip › Figure S4.pdf]

F1-measure

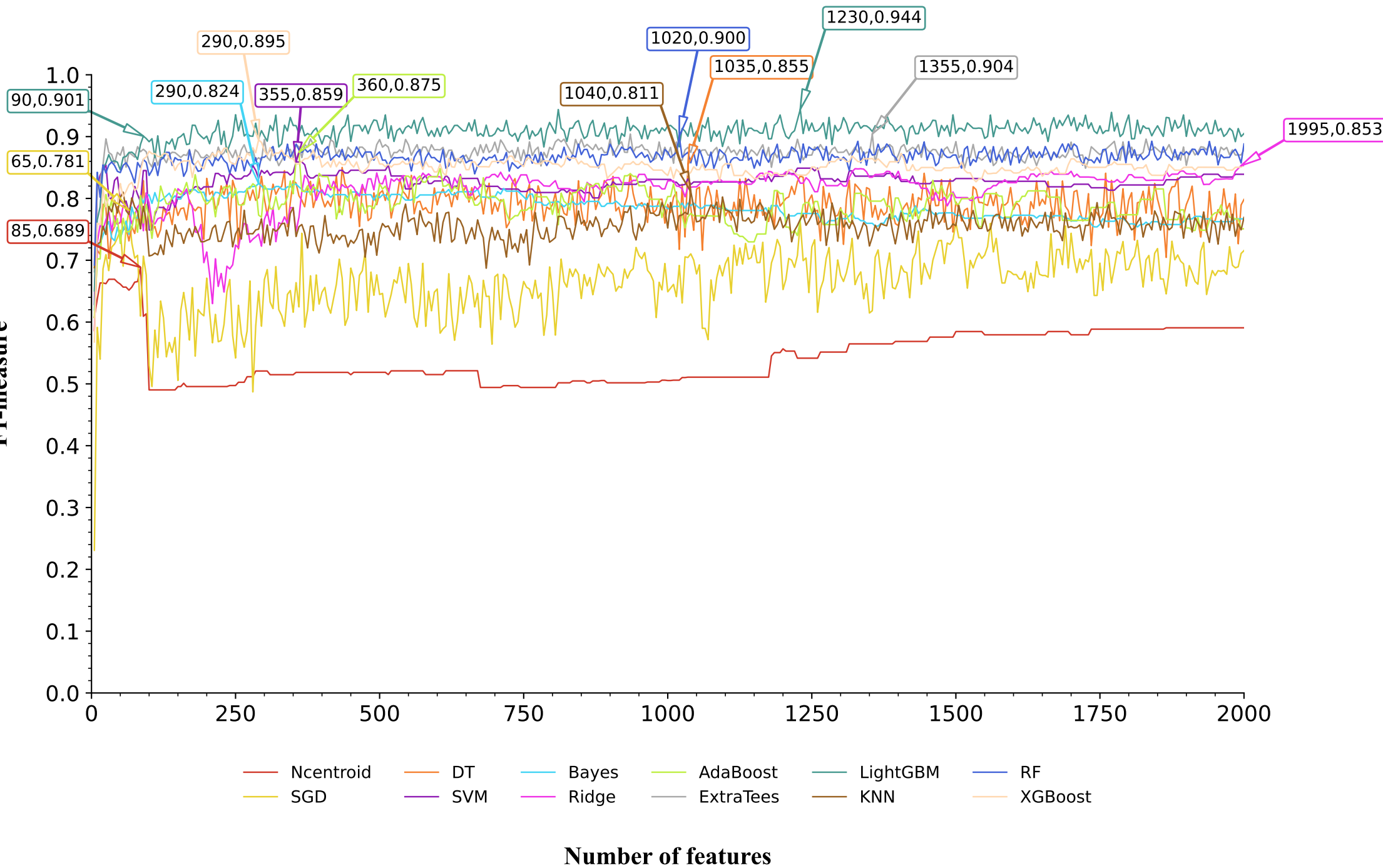

Supplement: Supplementary file 1 [file life-15-01039-s001.zip › Figure S5.pdf]

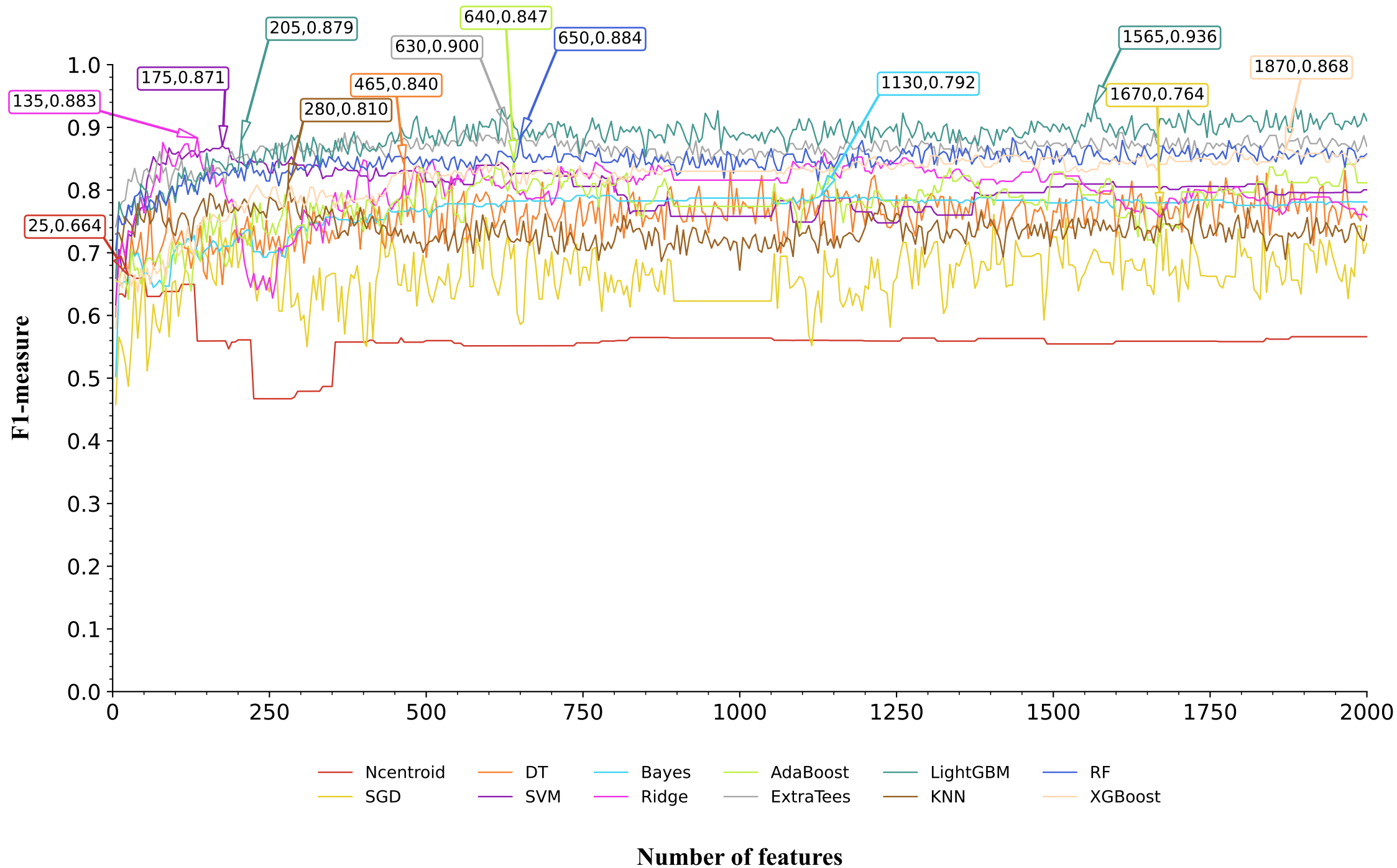

Supplement: Supplementary file 1 [file life-15-01039-s001.zip › Figure S6.pdf]

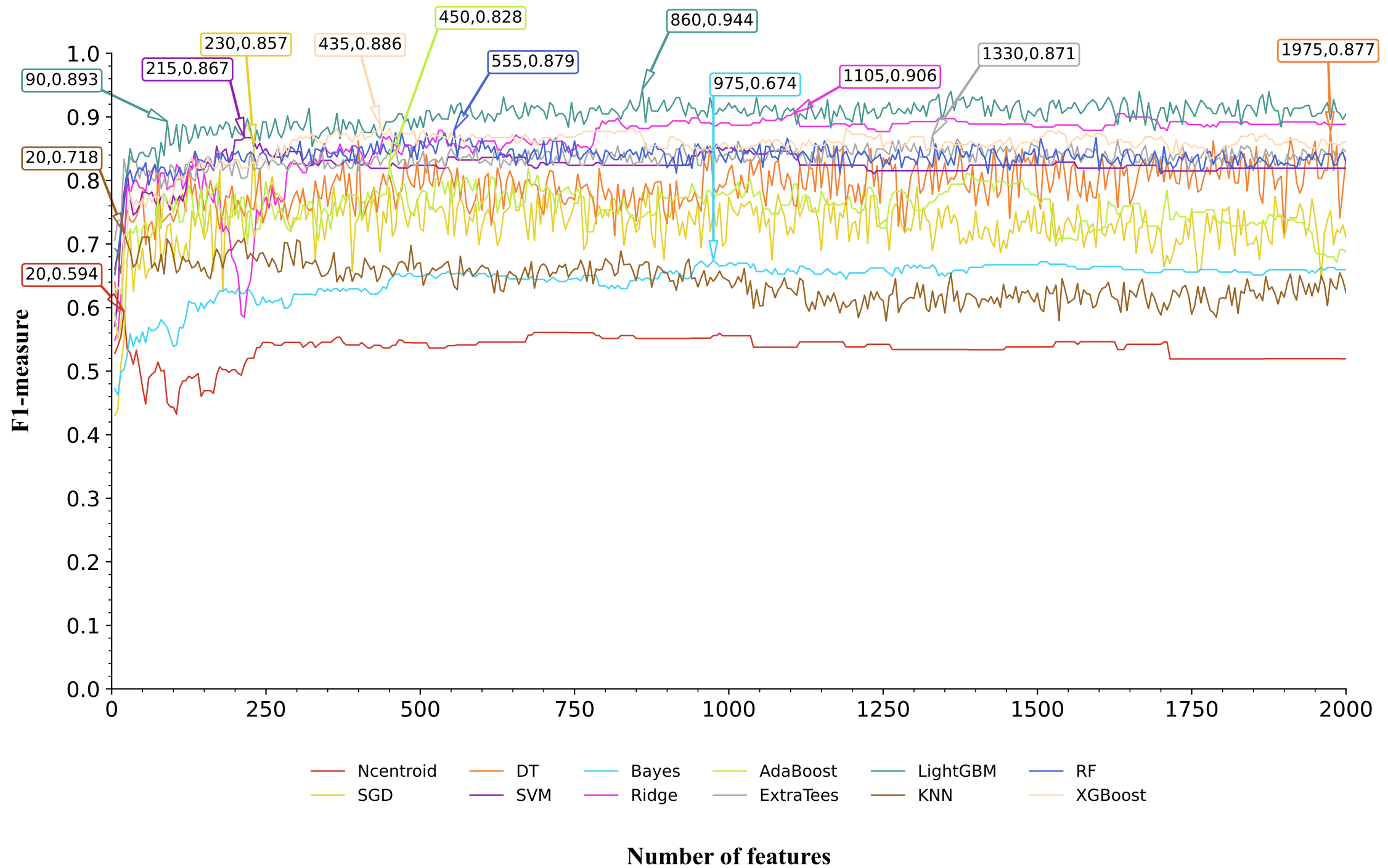

Supplement: Supplementary file 1 [file life-15-01039-s001.zip › Figure S7.pdf]

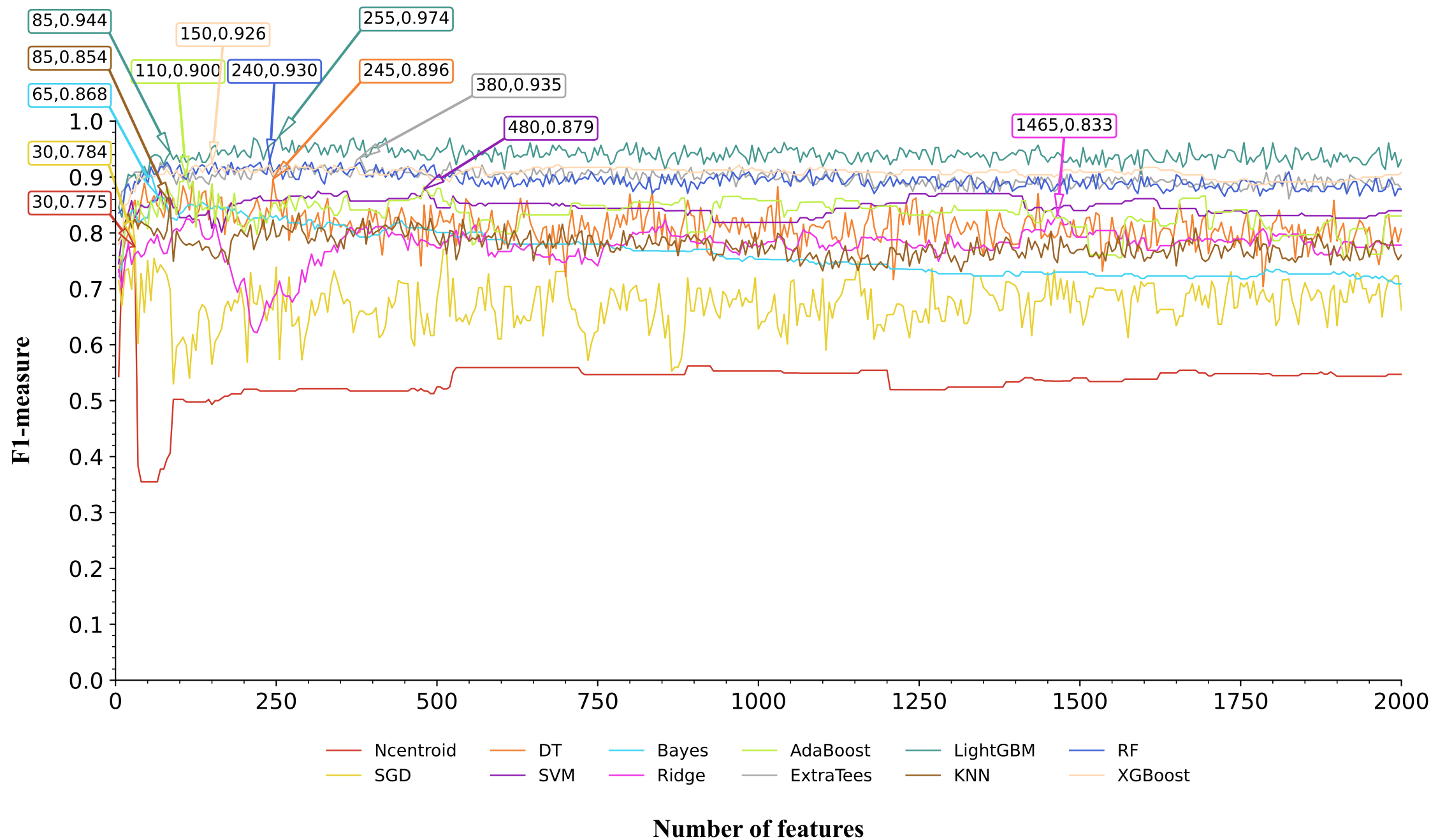

Supplement: Supplementary file 1 [file life-15-01039-s001.zip › Figure S8.pdf]
